# Body Composition and Intradialytic Exercise in Kidney Disease: A Combined Analysis of the PEDAL and CYCLE‐HD Randomised Controlled Trials

**DOI:** 10.1002/jcsm.13748

**Published:** 2025-03-03

**Authors:** Khai Ping Ng, Jamie H. Macdonald, Robin Young, Daniel S. March, Matthew P. M. Graham‐Brown, Thomas H. Mercer, Sharlene Greenwood, James O. Burton, Indranil Dasgupta

**Affiliations:** ^1^ Renal Medicine Royal Derby Hospital Derby UK; ^2^ Institute for Applied Human Physiology Bangor University Bangor UK; ^3^ Robertson Centre for Biostatistics Glasgow UK; ^4^ Department of Cardiovascular Sciences University of Leicester Leicester UK; ^5^ Centre for Health, Activity and Rehabilitation Research Queen Margaret University Edinburgh UK; ^6^ Department of Renal Medicine King's College Hospital NHS Trust London UK; ^7^ Renal Sciences, Faculty of Life Sciences and Medicine King's College London London UK; ^8^ Renal Medicine Birmingham Heartlands Hospital Birmingham UK; ^9^ Warwick Medical School University of Warwick Coventry UK

**Keywords:** BMI, fat mass, kidney diseases, muscle mass, myopenia, obesity, renal replacement therapy, sarcopenia

## Abstract

**Background:**

Haemodialysis patients are at high risk of myopenic obesity, necessitating effective nutritional status monitoring and intervention strategies. This combined analysis of two clinical trials (PEDAL trial and CYCLE‐HD study) aimed to (i) determine the clinical utility of body mass index (BMI) in comparison to fat tissue index (FTI) and lean tissue index (LTI) and (ii) assess the effect of a 6‐month intradialytic exercise intervention compared to usual care on FTI and LTI.

**Methods:**

A priori secondary endpoints in both trials included BMI, FTI and LTI. BMI was classified by World Health Organisation definitions (underweight, healthy, overweight or obese). FTI and LTI were determined by Bioelectrical Impedance Spectroscopy and classified by previous research evidence (FTI of 4–15 kg/m^2^ and LTI of 15–20 kg/m^2^ being associated with best survival). For aim (i), BMI was compared to FTI and LTI by correlation and classification. For aim (ii), changes over 6 months in FTI and LTI were compared between exercise intervention and control groups.

**Results:**

Across both studies, 298 and 209 participants had bioelectrical impedance spectroscopy measurement at baseline and 6 months, respectively. Mean (SD) age: 58 (15) years; BMI: 28.2(6.3) kg/m^2^; male: 65%. At baseline, only 47 of 298 participants (16%) had an FTI and LTI associated with best survival. BMI correlated with FTI (r = 0.79; *p* < 0.0001). However, 34% of participants were misclassified by BMI (e.g., 9% of patients were classified as obese by BMI yet FTI revealed their body composition was healthy). BMI did not correlate with LTI (*p* = 0.15), and 86% of participants were misclassified by BMI (e.g., 73% of patients were classified as healthy, overweight or obese by BMI yet LTI revealed they were myopenic). There was no difference between exercise intervention and control groups in mean change (95% CI) over 6 months for LTI (−0.3 [−1.1 to 0.4] kg/m^2^; *p* = 0.4) or FTI (0.2 [−0.7 to 1.0] kg/m^2^; *p* = 0.7).

**Conclusions:**

Worryingly, only a minority (16%) of haemodialysis patients had both LTI and FTI within the range associated with best survival. Body composition misclassification using conventional BMI cut‐offs was common: despite having healthy, overweight or even obese BMI, the majority (73%) of patients had hidden myopenia according to LTI. Six months of intradialytic aerobic exercise did not improve body composition. This study identified that common measures of nutritional status in haemodialysis patients such as BMI are misleading and that interventions other than intradialytic cycling are urgently required to target myopenic obesity.

## 1 Introduction

Myopenic obesity, defined as a state of decreased muscle mass and increased fat mass, is common in patients with chronic kidney disease [1]. A longitudinal study of incident haemodialysis patients reported increment of both body mass index (BMI) and fat tissue index (FTI), but a decrease in lean tissue index (LTI) over 24 months [2]. Unlike in the general population, BMI is inversely associated with survival in haemodialysis patients [3]. Data from the United States Renal Data System (USRDS) reported 6% greater risk of cardiovascular disease with each one‐unit decrement in BMI [4]. However, the relative contribution of lean tissue and fat tissue in affording this protection is not clearly defined.

A large international study of incident haemodialysis patients (*n* = 31 955) showed that LTI is a much more informative predictor of all‐cause mortality than FTI, with survival progressively reduced from the highest to the lowest quartile of LTI [5]. Study with surrogate markers of lean tissue and fat tissue also suggests that lean tissue is more protective than fat tissue [6]. Nevertheless, in a cohort of 162 Japanese people receiving haemodialysis, both FTI and LTI were reported to be independently, inversely associated with all‐cause mortality [7]. A study by the Monitoring Dialysis Outcomes (MONDO) initiative of 37 345 prevalent haemodialysis patients found that both LTI and FTI within the normal range (10th to 90th percentile) were associated with best survival [8].

Patients with kidney failure on haemodialysis are at high risk of myopenic obesity due to several catabolic factors and impairment of anabolic factors that operate in this group [9]. Therefore, there is need for effective nutritional status monitoring and intervention strategies in this population. The intradialytic period offers an ideal opportunity to implement exercise interventions in this population, and aerobic exercise is easier to implement intradialytically. The PEDAL trial (the PrEscription of intraDialytic exercise to improve quAlity of Life) [10] and the CYCLE‐HD study (a randomised controlled trial to investigate the effects of intradialytic cycling on left ventricular mass) [11] are two recent large interventional studies which further examined the clinical effectiveness of intradialytic cycling. Whilst 6 months of intradialytic cycling did not show improvement in health‐related quality of life in the PEDAL trial, the CYCLE‐HD study utilised a similar intervention and was found to significantly reduce left ventricular mass [10, 11]. Both trials included body composition as an a priori secondary endpoint and thus provide a unique opportunity to investigate body composition in a large data set. The aims of the present investigation were to combine data from the PEDAL and CYCLE‐HD trials to (i) determine the clinical utility of commonly used markers of nutritional status such as BMI and body mass change in comparison to LTI and FTI, as measured by bioelectrical impedance spectroscopy (BIS), and (ii) assess the effect of a 6‐month intradialytic exercise intervention compared to usual care on LTI and FTI.

## 2 Methods

The PEDAL study was a pragmatic multicentre randomised controlled trial in 335 UK haemodialysis patients primarily aimed to evaluate the effectiveness of a 6‐month intradialytic exercise programme on quality of life and physical function compared with usual care [10]. The CYCLE‐HD study was an open‐label, blinded endpoint, cluster randomised trial of 130 haemodialysis patients primarily aimed to evaluate the effect of intradialytic exercise programme on left ventricular mass [11]. The interventions are described in detail elsewhere [10, 11]. In summary, both studies implemented a 6‐month progressive intradialytic cycling programme, using specially adapted and calibrated exercise cycles, three times a week during dialysis, aiming for 30 min continuous cycling. Participants' age, BMI, body mass and diagnosis of diabetes mellitus were comparable between the two studies (Table S1).

Body composition measurement by BIS was an a priori secondary endpoint in both studies. The Body Composition Monitor (Fresenius Medical Care) was chosen as a well‐validated and widely used BIS‐based device [12]. BIS measurements were performed pre‐dialysis. Specifically, LTI and FTI were derived by dividing lean and fat tissue mass by height squared, respectively, data generated by the device [13]. Over‐hydration (OH) was provided by the device, being calculated from the difference between the normal expected extra cellular water and the actual extra cellular water. Of the participants in both studies, 298 had BIS measurement at baseline, whilst 209 had at least one BIS measurement completed at both baseline and 6 months (*n* = 111 from PEDAL; *n* = 98 from CYCLE‐HD). We combined the data from both studies for analysis. Baseline demographic data including age, sex, ethnicity, dialysis vintage, diagnosis of diabetes mellitus, body mass, BMI, pre‐dialysis systolic and diastolic blood pressure, serum albumin, haemoglobin, ferritin, calcium, phosphate, parathyroid hormone concentrations and weekly erythropoietin dose were recorded, as previously described [10, 11].

Compliance to exercise was calculated as the percentage of exercise sessions participants completed out of the total prescribed for the 6‐month intervention period. Compliance to a session was assumed when a diary entry was complete and included data for duration and rating of perceived exertion and a measure of power output or its surrogate (speed, gear and distance or energy expenditure).

### 2.1 Data Analysis

To determine the clinical utility of body composition measures, BMI was first classified according to World Health Organisation criteria: underweight: < 18.5 kg/m^2^; healthy weight: 18.5–24.9 kg/m^2^; overweight: 25.0–29.9 kg/m^2^; obese: ≥ 30.0 kg/m^2^. Body composition was then classified according to survival cut off values as per the MONDO study [2]: LTI values between 15.0 and 19.9 kg/m^2^ and FTI values between 4.0 and 14.9 kg/m^2^ were assumed to confer the best survival advantage and defined as healthy, with values below or above this range being defined as myopenia or myohypertrophy for LTI and under‐nourished or over‐nourished for FTI, respectively. Comparisons were then made between participant's BMI or body mass change and body composition to determine whether misclassification of body composition occurred if BMI or body mass change were relied upon alone. Note that these comparisons were made to determine the limitations of BMI and body mass change as markers of nutritional status, rather than to determine whether LTI or FTI are worthy therapeutic goals in themselves. Note that the sample population is representative of the target population; therefore, there were a relatively small number of participants in certain categories (e.g., underweight by BMI). Consequently, statistical comparisons were not attempted for this aim of the study to avoid issues with insufficient power.

Continuous variables are summarised by mean and standard deviation (SD), or median and interquartile range (IQR), unless stated. Categorical variables are summarised by *n* (%). The 6‐month changes in LTI, FTI and OH are analysed using linear regression adjusting for study (PEDAL or CYCLE‐HD), baseline value, age, sex and group allocation (exercise or control). All analyses were conducted in R v4.1.1. No corrections were made for multiple comparisons. *p* < 0.05 was considered significant.

## 3 Results

### 3.1 BMI Compared to LTI and FTI in Haemodialysis Population

Baseline demographic and clinical data are provided in Table 1. Comparing participants who provided baseline BIS data only, with participants who provided baseline and 6‐month BIS data, suggested participants were generally similar. Only parathyroid hormone was notable different, being lower in participants who provided baseline and 6‐month BIS data.

**TABLE 1:** Baseline demographic and clinical data for all participants.

	Participants with baseline BIS (*n* = 298)	Participants with baseline and 6‐month BIS (*n* = 209)	Exercise group (*n* = 99)	Usual care group (*n* = 110)
Age (years)	58 (15)	59 (15)	57 (15)	60 (14)
Male sex, *n* (%)	194 (65)	139 (67)	61 (62)	78 (71)
White ethnicity, *n* (%)	134 (45)	92 (44)	48 (49)	44 (40)
Dialysis vintage, median (IQR) (years)	1.3 (0.5, 3.4)	1.2 (0.5, 2.8)	1.3 (0.5, 2.6)	1.2 (0.4, 2.8)
Diabetes mellitus, *n* (%)	118 (40)	82 (39)	34 (34)	44 (48)
Body mass index (kg/m^2^)	28.2 (6.3)	28.1 (6.0)	28.0 (5.9)	28.3 (6.0)
Underweight by BMI, *n* (%)^a^	5 (2)	1 (2)	1 (1)	1 (1)
Healthy weight by BMI, *n* (%)^a^	96 (32)	67 (32)	32 (32)	32 (35)
Overweight by BMI, *n* (%)^a^	102 (34)	80 (38)	39 (39)	37 (42)
Obese by BMI, *n* (%)^a^	95 (32)	60 (29)	27 (27)	30 (33)
Body mass (kg)	79.6 (19.6)	79.8 (19.3)	78.9 (17.7)	80.6 (20.7)
Systolic blood pressure (mmHg)	138 (21)	140 (21)	138 (21)	141 (21)
Diastolic blood pressure (mmHg)	75 (14)	75 (14)	76 (14)	74 (13)
Serum albumin (g/L)	37 (5)	36 (5)	37 (5)	36 (5)
Haemoglobin (g/L)	110 (15)	110 (15)	111 (14)	110 (16)
Ferritin (μg/L)	283 (161)	291 (158)	280 (154)	301 (163)
Parathyroid hormone (pmol/L), median (IQR)	109 (28, 402)	91 (27, 402)	85 (26, 430)	109 (30, 333)
EPO weekly dose (unit), median (IQR)	6000 (1000, 9000)	6000 (1000, 9000)	5000 (0, 9000)	6000 (4000, 9000)
Quality of life (EQ‐5D‐5L score)	0.7 (0.3)	0.7 (0.3)	0.7 (0.3)	0.7 (0.3)
Lean tissue index (kg/m^2^)	12.8 (3.6)	12.8 (3.6)	12.7 (3.7)	12.9 (3.4)
Fat tissue index (kg/m^2^)	13.5 (7.0)	13.2 (6.1)	13.1 (6.4)	13.3 (5.9)
Overhydration index (kg/m^2^)	0.7 (2.2)	0.8 (1.9)	0.7 (1.9)	0.9 (1.8)

*Note:* Data are mean (SD) unless stated.

Abbreviations: BIS, bioelectrical impedance spectroscopy; BMI, body mass index; EPO, erythropoietin.

^a^ World Health Organisation criteria were used to define BMI as follows: underweight: < 18.5 kg/m^2^; healthy weight: 18.5–24.9 kg/m^2^; overweight: 25.0–29.9 kg/m^2^; obese: ≥ 30.0 kg/m^2^.

**FIGURE 1:**
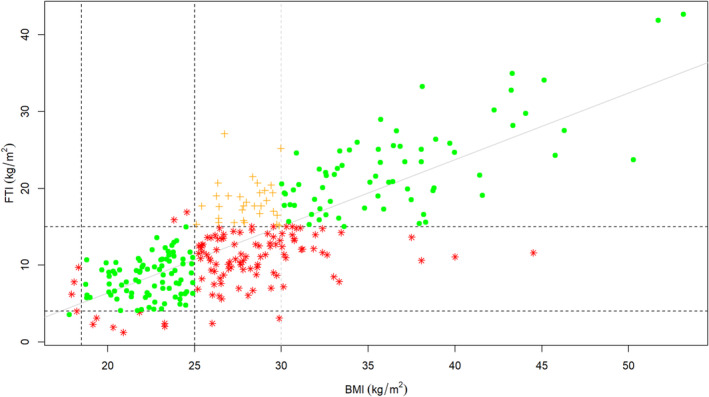
Relationship between body mass index and fat tissue index. BMI, body mass index; FTI, fat tissue index. Vertical lines, from left to right: World Health Organisation BMI criteria to define underweight (< 18.5 kg/m^2^); overweight (≥ 25 kg/m^2^); and obese (≥ 30 kg/m^2^) patients, respectively. Horizontal lines, from bottom to top: MONDO study [10] FTI survival cut off values to define under‐nourished (< 4 kg/m^2^) and over‐nourished (> 15 kg/m^2^) patients, respectively. Pearson's correlation coefficient *r* = 0.79; *p* < 0.0001; *n* = 298. Green dots (·): agreement between BMI classification and FTI classification of nutritional status. Amber plus signs (+): agreement between body mass index classification and fat tissue index classification of nutritional status. However, overweight by body mass index classification (but not obese, and thus unlikely to trigger clinical intervention) despite being over‐nourished by fat tissue index. Red stars (*): disagreement between body mass index classification and fat tissue index classification of nutritional status.

In terms of FTI, 186 of 298 participants (63%) were healthy (4–15 kg/m^2^), 10 participants (3%) were under‐nourished (values below 4 kg/m^2^) whilst 102 participants (34%) were over‐nourished (values above 15 kg/m^2^) (Figure 1 and Table 2). BMI significantly correlated with FTI (Figure 1). Of the 298 participants, 196 (66%) showed good agreement between BMI and FTI classifications of nutritional status. However, 102 participants (34%) were misclassified by BMI (Figure 1 and Table 2). Note that 95 participants (32%) were overweight or obese by BMI, yet FTI revealed their body composition was healthy.

**TABLE 2:** Distribution of body mass index and fat tissue index.

Body mass index (kg/m^2^)	Fat tissue index (kg/m^2^)	Number of participants (*n* = 298)	%
< 18.5	0.0–3.9	1	0.3
	**4.0**–**14.9**	**4**	**1.3**
	**≥ 15.0**	**0**	**0**
18.5–24.9	**0.0**–**3.9**	**7**	**2.4**
	4.0–14.9	87	29.2
	**≥ 15.0**	**2**	**0.7**
25–29.9	**0.0**–**3.9**	**2**	**0.7**
	**4.0**–**14.9**	**69**	**23.2**
	*≥ 15.0*	*31*	*10.4*
≥ 30.0	**0.0**–**3.9**	**0**	**0**
	**4.0**–**14.9**	**26**	**8.7**
	≥ 15.0	69	23.2

*Note:* The World Health Organisation defines body mass index as underweight: < 18.5 kg/m^2^; healthy weight: 18.5–24.9 kg/m^2^; overweight: 25.0–29.9 kg/m^2^; obese: ≥ 30 kg/m^2^. From the MONDO study [2], FTI values between 4.0 and 14.9 kg/m^2^ were assumed to confer the best survival advantage and defined as healthy, with values below or above this range being defined as under‐nourished or over‐nourished, respectively. Green rows/normal font: agreement between body mass index classification and fat tissue index classification of nutritional status. Amber row/italic font: agreement between body mass index classification and fat tissue index classification of nutritional status. However, overweight by body mass index classification (but not obese, and thus unlikely to trigger clinical intervention) despite being over‐nourished by fat tissue index. Red rows/bold font: disagreement between body mass index classification and fat tissue index classification of nutritional status.

**FIGURE 2:**
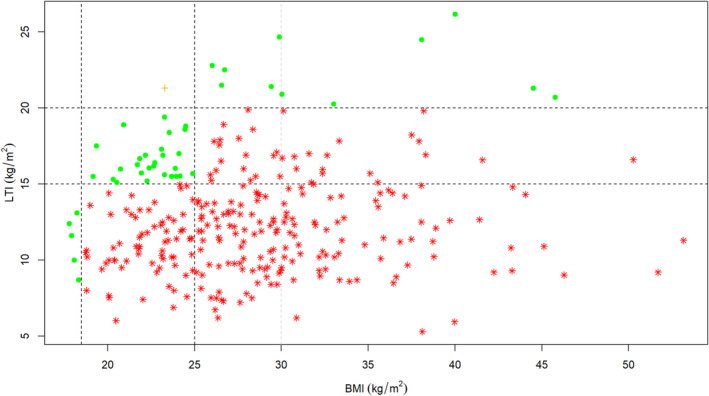
Relationship between body mass index and lean tissue index. BMI, body mass index; LTI, lean tissue index. Vertical lines, from left to right: World Health Organisation BMI criteria to define underweight (< 18.5 kg/m^2^); overweight (≥ 25 kg/m^2^); and obese (≥ 30 kg/m^2^) patients, respectively. Horizontal lines, from bottom to top: MONDO study [2] LTI survival cut off values to define myopenia (< 15 kg/m^2^) and myohypertrophy (> 20 kg/m^2^) patients, respectively. Pearson's correlation coefficient *r* = 0.08; *p* = 0.15; *n* = 298. Green dots (·): agreement between body mass index classification and lean tissue index classification of nutritional status. Orange plus signs (+): disagreement between body mass index classification and lean tissue index classification of nutritional status. However, healthy by body mass index classification and myohypertrophy suggests nutritional status is unlikely to be of clinical concern. Red stars (*): disagreement between body mass index classification and lean tissue index classification of nutritional status.

In terms of LTI, 63 of 298 participants (21%) were healthy (15–20 kg/m^2^), 223 participants (75%) were myopenic (values below 15 kg/m^2^), whilst 12 participants (4%) were myohypertrophic (values above 20 kg/m^2^) (Figure 2 and Table 3). There was no correlation between LTI and BMI in this haemodialysis cohort (Figure 2), and of the 298 participants, only 41 (14%) showed good agreement between BMI and LTI classifications of nutritional status. Thus, 257 participants (86%) were misclassified by BMI (Figure 2 and Table 3). Note that 217 participants (73%) were classified as healthy, overweight or obese by BMI, yet LTI revealed they were myopenic.

**TABLE 3:** Distribution of body mass index and lean tissue index.

Body mass index (kg/m^2^)	Lean tissue index (kg/m^2^)	Number of participants (*n* = 298)	%
< 18.5	0.0–14.9	5	1.7
	**15.0**–**19.9**	**0**	**0**
	**≥ 20.0**	**0**	**0**
18.5–24.9	**0.0**–**14.9**	**68**	**22.8**
	15.0–19.9	27	9.1
	*≥ 20.0*	*1*	*0.3*
25–29.9	**0.0**–**14.9**	**79**	**26.5**
	**15.0**–**19.9**	**18**	**6.0**
	≥ 20.0	5	1.7
≥ 30.0	**0.0**–**14.9**	**71**	**23.8**
	**15.0**–**19.9**	**18**	**6**
	≥ 20.0	6	2

*Note:* The World Health Organisation defines body mass index as underweight: < 18.5 kg/m^2^; healthy weight: 18.5–24.9 kg/m^2^; overweight: 25.0–29.9 kg/m^2^; obese: ≥ 30 kg/m^2^. From the MONDO study [2], LTI values between 15.0 and 19.9 kg/m^2^ were assumed to confer the best survival advantage and defined as healthy, with values below or above this range being defined as myopenia and myohypertrophy, respectively. Green rows/normal font: agreement between body mass index classification and lean tissue index classification of nutritional status. Orange row/italic font: disagreement between body mass index classification and lean tissue index classification of nutritional status. However, healthy by body mass index classification and myohypertrophy suggests nutritional status is unlikely to be of clinical concern. Red rows/bold font: disagreement between body mass index classification and lean tissue index classification of nutritional status.

**FIGURE 3:**
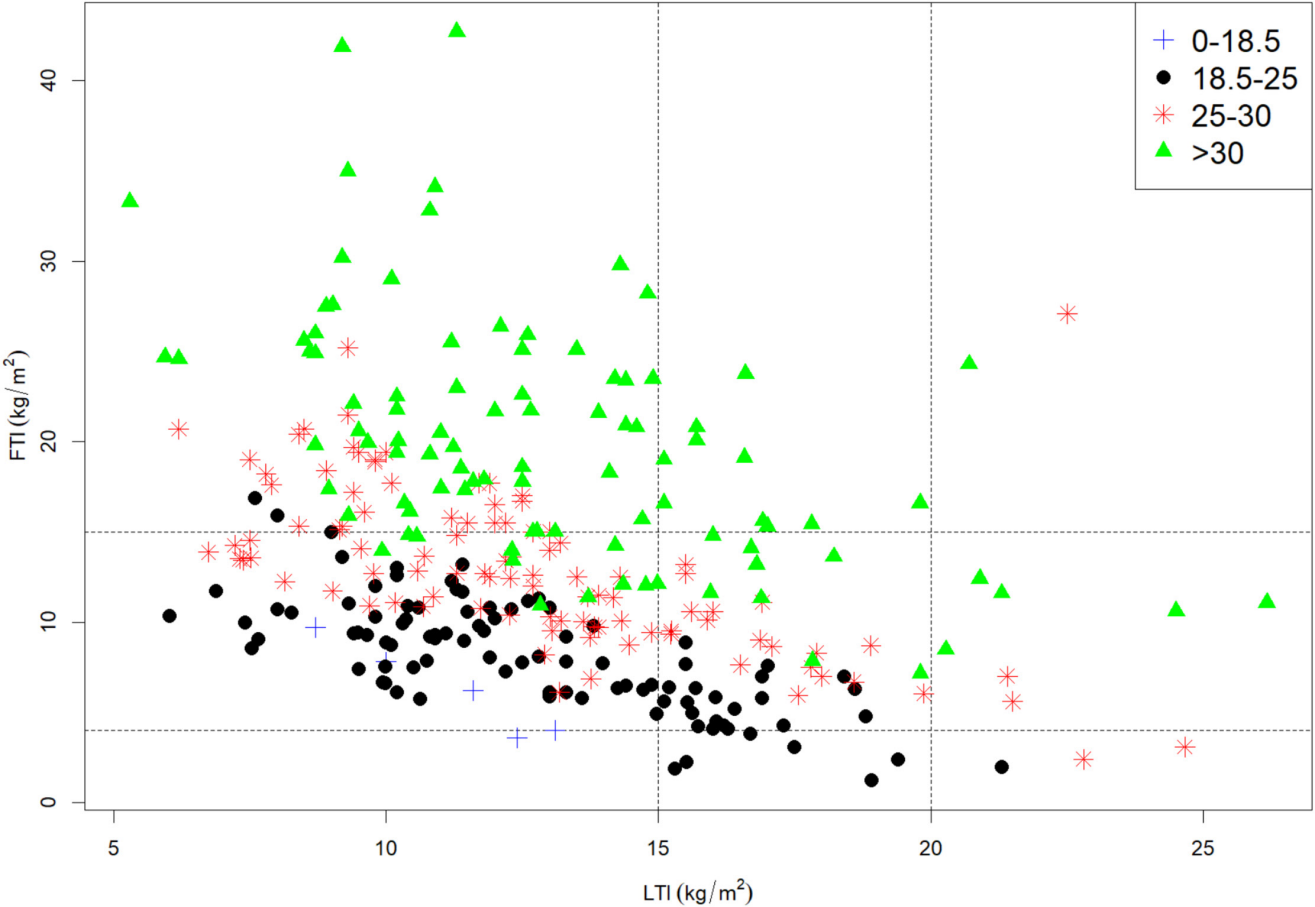
Relationship between body mass index, fat tissue index and lean tissue index. Position on the plot denotes LTI classification (horizontal axis) and FTI classification (vertical axis), defined as per the MONDO study [2] survival cut off values. Symbols denote BMI classification, defined as per World Health Organisation criteria. Patients who are in the healthy range for both LTI and FTI are shown in the central section of the plot. Patients who have an LTI below the recommended range are shown in the left lower, left middle and left upper sections. Patients who have an LTI above the recommended range are shown in the right lower, right middle and right upper sections. Patients who have an FTI above the recommended range are shown in the left upper, middle upper and right upper sections. Patients who have an FTI below the recommended range are shown in the left lower, middle lower and right lower sections. Blue plus signs (+), underweight by BMI; black dots (·), healthy weight; red stars (*), overweight; green triangles (▲), obese. *n* = 298.

Given the potential interaction between LTI and FTI, we evaluated the relationship amongst the three factors: BMI, LTI and FTI (Figure 3 and Table S2). Of note, 90 of 298 participants (30%) were myopenic by LTI (values below 15 kg/m^2^) but over‐nourished by FTI (values above 15 kg/m^2^), consistent with the condition of myopenic obesity. Only 47 of 298 participants (16%) had both LTI and FTI measurements within the healthy range (LTI values between 15 and 20 kg/m^2^ and FTI values between 4 and 15 kg/m^2^). This is in comparison to BMI measurement whereby 96 of 298 participants (32%) were categorised within the healthy range (BMI values between 18.5 and 24.9 kg/m^2^). Of the 47 participants with healthy LTI and FTI, 21 (7%) had healthy BMI, but 18 (6%) and 8 (3%) of participants had BMI classified as overweight or obese, respectively. Also of particular interest are the high proportion of the individuals (71 of 298, 24%) who had high BMI (values above 30 kg/m^2^) yet low LTI (values below 15 kg/m^2^), consistent with the condition of hidden myopenia.

**FIGURE 4:**
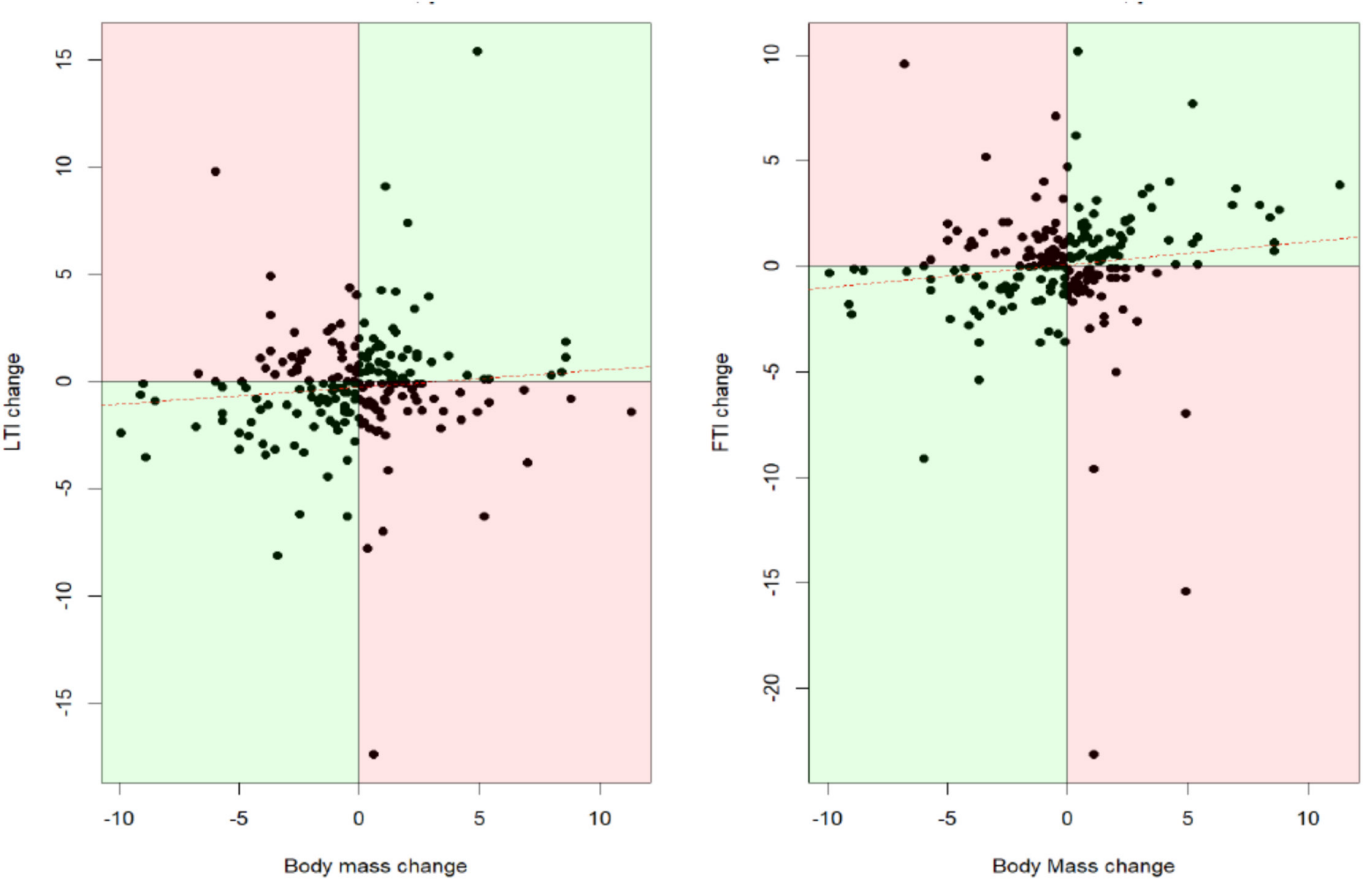
Change in body mass from baseline to 6 months compared to change in lean tissue index and change in fat tissue index. Green shading denotes agreement between body mass change and body composition parameter. Red shading denotes disagreement between body mass change and body composition parameter. Left panel: correlation between body mass change (kg) and LTI (lean tissue index) change (kg/m^2^): *r* = 0.09; *p* = 0.18. Right panel: correlation between weight change and FTI (fat tissue index) change (kg/m^2^): *r* = 0.11; *p* = 0.10. *n* = 209.

As well as BMI, body mass change is often used clinically as a marker of nutritional status. In both groups, during the 6‐month follow‐up period, there was no correlation between change of body mass and change of LTI or FTI (Figure 4). Of note, 54 of 209 participants (26%) and 39 of 209 participants (19%) increased their body mass but decreased their LTI or FTI, respectively (bottom right quadrant in each plot, Figure 4). Conversely, 36 of 209 participants (18%) and 45 of 209 participants (22%) decreased their body mass but increased their LTI or FTI, respectively (top left quadrant in each plot, Figure 4).

### 3.2 Effect of a 6‐Month Intradialytic Exercise Intervention on Body Composition

In total, 209 participants who had both baseline and follow‐up data for bioimpedance measurements were included in the intervention analysis: 110 received usual care, and 99 were assigned to intradialytic cycling in addition to usual care. The usual care group had slightly more male participants, slightly fewer white participants, slightly more participants with diabetes mellitus and higher parathyroid hormone concentration, but otherwise were well‐matched to the exercise intervention group (Table 1).

At baseline, the LTI, FTI and OH as measured by BIS were comparable between the groups (Table 1). There were no significant differences in body composition change scores over 6 months between exercise intervention and usual care groups (Table 4).

**TABLE 4:** Difference in body composition change scores over 6 months between intervention and usual care groups.

	Compliance to exercise	No. of participants (*n* = 209)		Difference in change from baseline to 6 months	95% confidence interval	*p* value
		Exercise intervention	Usual care			
LTI (kg/m^2^)	All participants	97	108	0.33	−1.08 to 0.41	0.4
	Compliance ≥ 30%	75	108	0.63	−1.47 to 0.20	0.1
	Compliance ≥ 50%	60	108	0.39	−1.26 to 0.48	0.4
	Compliance ≥ 70%	37	108	0.26	−1.30 to 0.77	0.6
OH (L)	All participants	99	110	−0.05	−0.41 to 0.51	0.8
	Compliance ≥ 30%	77	110	−0.15	−0.35 to 0.65	0.6
	Compliance ≥ 50%	62	110	−0.10	−0.43 to 0.63	0.7
	Compliance ≥ 70%	39	110	−0.24	−0.42 to 0.89	0.5
FTI (kg/m^2^)	All participants	97	107	−0.16	−0.69 to 1.00	0.7
	Compliance ≥ 30%	75	107	−0.32	−0.61 to 1.25	0.5
	Compliance ≥ 50%	60	107	−0.10	−0.87 to 1.07	0.8
	Compliance ≥ 70%	37	107	0.12	−1.29 to 1.06	0.8

*Note:* Difference in change = Exercise intervention change minus usual care change. Grey shading denotes primary analyses; unshaded denotes sensitivity analyses considering compliance. Compliance to exercise was calculated as the percentage of exercise sessions participants completed out of the total prescribed for the 6‐month intervention period.

Abbreviations: FTI: fat tissue index; LTI: lean tissue index; OH: over‐hydration.

Amongst the exercise intervention group, less than half of the participants (39 of 99, 43%) complied with ≥ 70% of the total cycling sessions, whilst 23 (26%), 15 (17%) and 13 (14%) complied with 50%–69%, 30%–49% and < 30% of the sessions, respectively. In a sensitivity analysis, the effect of compliance on LTI, FTI and OH was assessed. Consistent with the primary analysis, there was no significant differences in body composition change scores over 6 months between exercise intervention and usual care groups (Table 4), even in participants who were the most compliant. Visual inspection of 95% confidence intervals suggested this lack of a statistical effect was not due to the smaller sample size of the sensitivity analyses.

## 4 Discussion

This combined analysis of the PEDAL trial and CYCLE‐HD study data demonstrated that only a minority (16%) of haemodialysis patients had both LTI and FTI within the range previously demonstrated to be associated with best survival in haemodialysis patients [8], reiterating the importance of identifying effective nutritional status monitoring and intervention strategies for this population. Although BMI correlated with FTI, BMI did not correlate with LTI. Furthermore, when using BMI as an indicator of nutritional status (or obesity), 34% of patients were misclassified, and when using BMI as an indicator of nutritional status (or myopenia), 86% of patients were misclassified. Even body mass change over time failed to correctly identify increases in fat mass or decreases in lean mass in over a quarter of patients. These data highlight the issues of body composition misclassification using conventional BMI cut‐offs and body mass change in this population. Consequently, three quarters of participants were classified as healthy, overweight or obese by BMI yet were myopenic according to LTI, consistent with hidden myopenia. It was thus disappointing that the 6‐month intradialytic exercise intervention did not result in a significant improvement in body composition compared with usual care.

Body mass index is a commonly used clinical marker of body fat and nutritional status. It is often used as a metric when determining nutrition intervention and public health policies. In patients with end‐stage kidney disease (ESKD), it is used to assess suitability to receive kidney transplantation. At a population level, BMI is a reasonable marker of FTI and, in most people, correctly identifies under‐nourished, healthy and over‐nourished individuals. However, its accuracy in diagnosing obesity might be limited in men, in the elderly and amongst individuals with intermediate BMI ranges as it fails to discriminate between body fat and lean mass [14]. Moreover, the ‘obesity paradox’, whereby obesity appears to confer survival advantage in subpopulations of cardiovascular disease, cancer, diabetes, respiratory disease and renal disease [15], suggests that BMI fails to adequately capture the subtlety of body composition that is crucial in predicting relevant outcomes. In a large prospective cohort study of health professionals in the United States, the ‘obesity paradox’ was found to be largely explained by low lean body mass in the lower range of BMI, rather than low fat mass [16].

This study further highlights the fallacies of using BMI to assess the state of nourishment of an individual, especially in the haemodialysis population. In this study population at baseline, 2% had low BMI, 32% had normal BMI and 66% had BMI in overweight‐obese range. Such high prevalence of elevated BMI is representative of the population. Temporal trend of increasing BMI has been reported in haemodialysis population [17]. However, bioelectrical impedance spectroscopy showed that the prevalence of hidden myopenia was high; 73% of haemodialysis patients with normal, overweight or obese BMI were in fact myopenic by LTI. Furthermore, 30% of the total cohort were myopenic by LTI and over‐nourished by FTI, consistent with the condition of myopenic obesity. Thus, myopenic obesity in our representative population is slightly higher than commonly appreciated, as previous studies suggest prevalence varies from 2% to 23% [1].

Bioelectrical impedance spectroscopy is a simple, quick and non‐invasive assessment, which provides measurements of fat mass and lean mass. In the present cohort, using bioelectrical impedance identified 47 (16%) more patients than BMI who should be considered further for clinical intervention. More detailed information on body composition obtained via bioelectrical impedance spectroscopy thus provides an additional marker that can compensate for limitations in BMI, aiding diagnosis of nutritional issues, and also offers opportunities to better understand the complex relationship between body mass and mortality in haemodialysis population.

Relying on BMI alone, which does not distinguish between fat mass and lean mass, can lead to incorrect, and even harmful, nutritional advice being given. For instance, an obese haemodialysis patient with normal FTI may be put through the weight management service, wasting resource to try to reduce body and fat mass. This will increase patient burden, potentially restrict opportunity for kidney transplantation and increase the risk of poor outcome. By contrast, an obese person with normal or low LTI may be given weight reducing advice, causing loss of muscle mass, reducing functional capacity and increasing the risk of poor outcome. Equally, relying on the commonly used BMI cut‐off alone also results in some patients who have BMI within normal range but low LTI being missed, leading to a missed opportunity to address a modifiable risk of poor outcome [8].

However, LTI and FTI are relatively novel outcomes to assess nutritional status, and hence, it remains important to summarise studies investigating LTI and FTI as markers of survival. Low LTI was reported to be highly predictive of mortality in a haemodialysis cohort (*n* = 123) in China, independent of FTI and subjective global assessment [18]. Similarly, a cohort of 226 French haemodiafiltration patients reported lean body mass as predictor of long‐term survival [6]. Conversely, in a larger haemodialysis population (*n* = 697) in Portugal, low FTI was found to be useful predictors of mortality [19]. A longitudinal study of 535 haemodialysis patients observed independent association between low baseline percentage body fat and fat loss (≥ 1%) over time with higher mortality, even after adjustment for surrogates of muscle mass and inflammation. Nevertheless, a Japanese study of 162 haemodialysis patients concluded that higher FTI and/or higher LTI were independently associated with reduced all‐cause mortality [7]. Another study of 808 Japanese haemodialysis patients reported independent associated between higher LTI and reduced cardiovascular death as well as higher FTI and reduced non‐cardiovascular death [20]. Overall, the MONDO study of 37 345 dialysis patients represented one of the largest international cohort data on body composition and survival [8]. Whilst the MONDO consortium concluded that patients with both LTI and FTI in the 10th to 90th of a healthy population (15–20 and 4–15 kg/m^2^, respectively) confer best survival, significant interaction between FTI and LTI was noted, with higher FTI appearing to be protective in patients with low LTI [8]. To individualise clinical care and provide the best nutritional advice, both LTI and FTI must, therefore, be interpreted together and considered as additional markers of nutritional status rather than therapeutic goals in themselves.

Further illustration of the potential utility of LTI and FTI as markers of nutritional status, and of the caution required should LTI and FTI be used as a therapeutic goal per se, is provided in Figure 3. A significant proportion of the participants had LTI below 15 kg/m^2^ (left column) potentially requiring intervention to reverse protein wasting. This is most important for those in the left middle and left lower sections with normal or low FTI, who may benefit from dietary supplementation to increase energy intake, and anabolic interventions such as progressive resistance training. However, it should be done carefully for those with high FTI (left upper section) as simply increasing energy intake may be harmful; instead, an increase in protein intake and intervention with progressive resistance training would be preferable. Conversely, for participants with high FTI (more than 15 kg/m^2^, upper section of Figure 3) requiring intervention to reduce fat mass, those in the middle upper and right upper sections with normal or high LTI may benefit from simple energy restriction and aerobic physical activity. Conversely, weight loss should be promoted very carefully for patients in the left upper sections of the graph with low LTI to avoid exacerbating protein wasting. Relying on BMI alone would not allow such an individualisation of treatment and may lead to potentially harmful intervention.

Body mass change over time is another commonly used clinical marker of nutritional status. A strength of the present study is the inclusion of longitudinal follow up data, allowing the clinical utility of body mass change, LTI and FTI to be assessed over time. The study again highlighted the fallacy of relying on body mass change alone, as it misclassified some patients who had stable or increasing body mass yet had lost lean tissue and misclassified others who had stable or reducing body mass yet had gained fat tissue (Figure 4).

Protein wasting, high burden of pre‐existing comorbidities, recurrent hospitalisation and psychological stress often lead to myopenia and reduced physical exercise capacity amongst the dialysis population, both of which are associated with adverse clinical outcomes and poor quality of life [21]. As expected, nutritional support with or without exercise training has been the mainstay intervention aiming to optimise the physical functioning amongst this vulnerable group of patients.

In general, aerobic exercise improves cardiovascular endurance and reduces BMI and FTI, whilst progressive resistance training is the optimal mode of exercise to increase LTI [22]. Amongst aging general populations, resistance exercise has been found to improve lean tissue mass, especially with programmes utilising higher intensity exercise [23]. However, patients with kidney failure are often sedentary and deconditioned and have reduced physical exercise capacity. The threshold for the anabolic or anti‐catabolic effect of exercise on a sedentary haemodialysis population might therefore be lower compared to the general population. A previous systematic review and meta‐analysis of 20 randomised controlled trials, predominantly examining aerobic and combined (aerobic and resistance) exercise in haemodialysis patients, concluded that such interventions improved aerobic capacity, walking capacity and health‐related quality of life [24], with limited data on the effect of intradialytic aerobic exercise on body composition. Despite clear evidence of benefits of exercise, including on body composition, in the general population [25, 26], the combined data from the PEDAL trial and CYCLE‐HD study demonstrated no impact on body composition with 6‐month intradialytic cycling exercise training in haemodialysis patients. This lack of effect is consistent with a previous literature review of exercise training in haemodialysis population that concluded modest or inconsistent benefits [27], and a recent meta‐analysis reporting no effect of intradialytic exercise, on body composition [28].

Whilst this study focused on the effect of intradialytic exercise on body composition, the importance of physical function measurement should be acknowledged, especially when discussing LTI. The consensus from the European Working Group on Sarcopenia in Older People recommended using the presence of both low muscle mass and low muscle strength in diagnosing sarcopenia [29]. Study of older general populations has highlighted the discrepancy between muscle quality and quantity as low muscle strength was found to be independently associated with increased all‐cause mortality, regardless of muscle mass [30]. Although muscle strength was not recorded in the present study, physical function was. The lack of observed effect with PEDAL [10] and CYCLE‐HD [11] is in contrast to a recent multi‐centre, cluster randomised trial of 917 patients, the DiaTT study, which demonstrated that 12 months of intradialytic combined endurance and resistance exercise training significantly improved physical function (60‐s sit‐to‐stand test) compared to usual care [31].

There are several plausible explanations to the contrasting findings. First is the volume, intensity and location of the exercise. The DiaTT study included a more intensive progressive resistance training intervention (involving dumbbells), and a considerable period of the intervention (11–32 weeks) was completed at home (due to the pandemic) [31]. Presumably, the intensity of exercise was greater than that achieved in PEDAL and CYCLE‐HD. Relatedly, it is important to note that the overall adherence amongst the exercise groups in PEDAL and CYCLE‐HD was suboptimal, with more than half (57%) completing less than 70% of the prescribed exercise sessions. Despite adopting a progressive exercise plan and individualised training intensity in the PEDAL trial and CYCLE‐HD study, the majority of the participants did not achieve and/or maintain the prescribed frequency, volume and intensity of exercise. This is in contrast to DiaTT and the EXCITE randomised trial, which implemented 6‐month, personalised, home‐based, low‐intensity walking exercise programme amongst patients on dialysis, and reported improvement in physical function, presumably due to its delivery at home and higher overall adherence to the prescribed exercise sessions [32]. Symptoms of fatigue especially on dialysis day, shortness of breath and lacking of motivation are some of the common issues known to hinder exercise behaviours in the dialysis population. A systematic review of qualitative studies also highlighted disease distress, negative perceptions of exercise and environmental restriction as key barriers to exercise participation in haemodialysis patients [33]. We believe, on the basis of our analysis, that the high prevalence of myopenia was likely to have contributed to this. It is possible that the majority of patients on maintenance haemodialysis might be too deconditioned to adhere to standard exercise regime and any individualised exercise training will require a lower ‘start‐point’, longer ‘phase‐in’ period and more variation and flexibility in scheduling to encourage and sustain adherence. A survey of barriers to exercise in patients with ESKD reported that 73% preferred to exercise at home and 41% preferred the combination of aerobic and resistance training [34]. Digital health innovations may represent a potential pragmatic solution. The Kidney BEAM platform, a physical activity digital health intervention developed in response to the COVID‐19 pandemic, has recently been reported to be effective in enhancing health‐related quality of life in patients with chronic kidney disease in multi‐centre randomised controlled trial [35]. This remote, flexible and scalable intervention might therefore serve as a useful ‘start‐point’, ‘phase‐in’ and individualised exercise intervention.

Second, the pathogenesis of physical inactivity amongst the haemodialysis population is often multifactorial and inter‐related. In addition to exercise compliance, other factors are likely to be in play, as supported by our sensitivity analysis of exercise compliance that did not alter our conclusion of no effect of exercise intervention on body composition. The link between malnutrition, chronic systemic inflammation and atherosclerosis cardiovascular disease (MIA syndrome) is well‐recognised [36]. Cardiovascular disease and chronic inflammation, which are prevalent amongst patient on dialysis, play a significant role in perpetuating or even accentuating protein wasting and in turn contributes to low exercise capacity. Likewise, low exercise capacity, which often results in a sedentary lifestyle, often leads to worsened body composition and increased morbidity and mortality risks. Both PEDAL and CYCLE‐HD studies did not include additional, parallel dietetic intervention, which might also contribute to the lack of changes in body composition findings despite exercise programme. With such a close, bidirectional relationship between protein wasting and low exercise capacity, any strategy for improvement will require a combination of individualised nutritional support based on body composition and exercise intervention tailored to patients' individual needs, as well as medical optimisation of underlying co‐morbidities, fluid status and dialysis clearance.

Third, we also considered if the participants' characteristics or duration of the intervention contributed to the lack of effect. Whilst no significant decrease in body composition in the 6‐month period in the exercise group could be the effect of intervention maintaining lean mass, this was not the case given a similar finding in the usual care group. Duration of intervention is often considered an important factor in outcome measures. A literature review by Clyne et al. summarised 14 studies of exercise training in patients on dialysis with only two with intervention longer than 6 months [37]. Whilst the DiaTT study demonstrated improvement with physical function following 12‐month endurance and resistance exercise intervention, EXCITE trial equally reported similar outcome with 6‐month low‐intensity intervention [31, 32]. Hence, the negative finding in our study was unlikely to be due to the study duration.

Notwithstanding exercise and nutrition interventions, several other therapy options have been proposed and trialled in attempt to improve body composition amongst patients with ESKD. Whilst meta‐analysis suggested correcting metabolic acidosis significantly improved muscle mass assessed by mid‐arm muscle circumference in non‐dialysis dependent chronic kidney disease population, the use of oral sodium bicarbonate supplement in a randomised study of 43 haemodialysis patients failed to conclude statistically significant benefit in improving lean mass but increased medical burden and pre‐dialysis blood pressure [38]. Another pilot study involving 33 maintenance haemodialysis patients suggested beneficial effects on preserving lean body mass with high‐volume online haemodiafiltration compared to conventional high‐flux haemodialysis [39]. Nevertheless, muscle mass preservation did not impact on muscle strength in this small, exploratory study.

In conclusion, this study highlighted issues of body composition misclassification using conventional BMI cut‐offs in haemodialysis patients. Only a minority of patients had both LTI and FTI within the range associated with best survival. Hidden myopenia is prevalent and 30% of our participants presented with myopenic obesity. A 6‐month intradialytic cycle exercise intervention did not improve body composition, suggesting alternative interventions, based on individual needs, are required to target fat and lean tissue mass and enhance survival of haemodialysis patients.

## Ethics Statement

The authors certify that this manuscript complies with the ethical guidelines for authorship and publishing in the *Journal of Cachexia, Sarcopenia and Muscle*. London Fulham Research Ethics Committee approved the PEDAL trial protocol (14/LO/1851), and the study was prospectively registered (ISRCTN N83508514). East Midlands Research Ethics Committee approved the CYCLE‐HD study protocol (14/EM/1190), and the study was prospectively registered (ISRCTN N11299707). All studies were performed in accordance with the ethical standards laid down in the 1964 Declaration of Helsinki. All participants gave their informed consent prior to their inclusion in the study.

## Conflicts of Interest

Khai Ping Ng, Jamie H. Macdonald, Robin Young, Daniel S. March, Matthew P. M. Graham‐Brown, Thomas H. Mercer, Sharlene Greenwood and Indranil Dasgupta declare no conflicts of interest.

James O. Burton is funded (Senior Investigator Award) by the National Institute for Health and Care Research (NIHR). The views expressed are those of the authors and not necessarily those of NIHR or the Department of Health and Social Care.

## Supporting information


**Table S1** Baseline characteristics of participants in PEDAL and CYCLE‐HD studies.
**Table S2** Distribution of body mass index, fat tissue index and lean tissue index (*n* = 298).
