# Supplementary material for: Body Composition and Intradialytic Exercise in Kidney Disease: A Combined Analysis of the PEDAL and CYCLE‐HD Randomised Controlled Trials
Source: J Cachexia Sarcopenia Muscle. 2025 Mar 3;16(2):e13748. doi: 10.1002/jcsm.13748 (PMC11873537; doi:10.1002/jcsm.13748)
Supplement: Supplementary file 1 — Table S1 Baseline characteristics of participants in PEDAL and CYCLE‐HD studies. Table S2 Distribution of body mass index, fat tissue index and lean tissue index (n = 298). [file JCSM-16-e13748-s001.docx]

**Supplementary Table 1:** Baseline characteristics of participants in PEDAL and CYCLE-HD studies.

|  | PEDAL study  (n=335) | CYCLE-HD study  (n=130) |
| --- | --- | --- |
| Age (years) | 59 (15) | 57 (15) |
| Male sex, n (%) | 207 (62) | 95 (73) |
| White ethnicity, n (%) | 169 (50) | 58 (45) |
| Diabetes mellitus, n (%) | 132 (39) | 49 (38) |
| Body mass index (kg/m^2^) | 28.7 (6.7) | 27.9 (5.9) |
| *Underweight by BMI, n (%) | 6 (1.8) | 1 (0.8) |
| *Healthy weight by BMI, n (%) | 97 (29) | 44 (34) |
| *Overweight by BMI, n (%) | 108 (32) | 47 (36) |
| *Obese by BMI, n (%) | 122 (37) | 38 (29) |
| Body weight (kg) | 81 (20) | 79 (19) |

Data are man (SD) unless stated.

**Supplementary Table 2:** Distribution of body mass index, fat tissue index and lean tissue index (n = 298).

|  | | **Body mass index (kg/m^2^)** | | | |
| --- | --- | --- | --- | --- | --- |
|  |  | < 18.5 | 18.5 to 24.9 | ≥ 25 to 29.9 | ≥ 30.0 |
| **Lean tissue index (kg/m^2^)** | **Fat tissue index**  **(kg/m^2^)** | **No. of patients** | | | |
| 0.0 to 14.9 | 0.0 to 3.9 | 1 | 0 | 0 | 0 |
|  | 4.0 to 14.9 | 4 | 66 | 49 | 13 |
|  | ≥15.0 | 0 | 2 | 30 | 58 |
| 15.0 to 19.9 | 0.0 to 3.9 | 0 | 6 | 0 | 0 |
|  | 4.0 to 14.9 | 0 | 21 | 18 | 8 |
|  | ≥15.0 | 0 | 0 | 0 | 10 |
| ≥ 20.0 | 0.0 to 3.9 | 0 | 1 | 2 | 0 |
|  | 4.0 to 14.9 | 0 | 0 | 2 | 5 |
|  | ≥15.0 | 0 | 0 | 1 | 1 |

Grey shading denotes healthy ranges for body mass index, fat tissue index and lean tissue index, as per World Health Organisation **and** MONDO study (2) definitions.
